# Histone H1 variant-specific lysine methylation by G9a/KMT1C and Glp1/KMT1D

**DOI:** 10.1186/1756-8935-3-7

**Published:** 2010-03-24

**Authors:** Thomas Weiss, Sonja Hergeth, Ulrike Zeissler, Annalisa Izzo, Philipp Tropberger, Barry M Zee, Miroslav Dundr, Benjamin A Garcia, Sylvain Daujat, Robert Schneider

**Affiliations:** 1 MPI for Immunobiology, Stübeweg 51, 79108 Freiburg, Germany; 2 Department of Molecular Biology, Princeton University, 415 Shultz Laboratory, Princeton, NJ 08540, USA; 3 Department of Cell Biology, Rosalind Franklin University, 3333 Green Bay Road, Chicago, IL 60064, USA

## Abstract

**Background:**

The linker histone H1 has a key role in establishing and maintaining higher order chromatin structure and in regulating gene expression. Mammals express up to 11 different H1 variants, with H1.2 and H1.4 being the predominant ones in most somatic cells. Like core histones, H1 has high levels of covalent modifications; however, the full set of modifications and their biological role are largely unknown.

**Results:**

In this study, we used a candidate screen to identify enzymes that methylate H1 and to map their corresponding methylation sites. We found that the histone lysine methyltransferases G9a/KMT1C and Glp1/KMT1D methylate H1.2 *in vitro *and *in vivo*, and we mapped this novel site to lysine 187 (H1.2K187) in the C-terminus of H1. This H1.2K187 methylation is variant-specific. The main target for methylation by G9a in H1.2, H1.3, H1.5 and H1.0 is in the C-terminus, whereas H1.4 is preferentially methylated at K26 (H1.4K26me) in the N-terminus. We found that the readout of these marks is different; H1.4K26me can recruit HP1, but H1.2K187me cannot. Likewise, JMJD2D/KDM4 only reverses H1.4K26 methylation, clearly distinguishing these two methylation sites. Further, in contrast to C-terminal H1 phosphorylation, H1.2K187 methylation level is steady throughout the cell cycle.

**Conclusions:**

We have characterised a novel methylation site in the C-terminus of H1 that is the target of G9a/Glp1 both *in vitro *and *in vivo*. To our knowledge, this is the first demonstration of variant-specific histone methylation by the same methyltransferases, but with differing downstream readers, thereby supporting the hypothesis of H1 variants having specific functions.

## Background

In eukaryotic cells, DNA is packaged into chromatin. The building block of chromatin is the nucleosomal core particle containing a histone octamer (formed by the histones H2A, H2B, H3 and H4) around which 147 bp of DNA (147 bp) are wrapped [1]. The linker histone H1 binds to the DNA between the nucleosomal core particles, and is essential to stabilise higher order chromatin structures [2].

Human cells possess up to 11 H1 variants, all consisting of a short N-terminal tail, a globular core domain and a C-terminal tail, making up approximately 50% of the whole protein [3,4]. H1.0 is mainly expressed in terminally differentiated cells. H1.1 expression has to date only been reported for a subset of tissues. H1.2 to H1.5 are expressed in almost all cells, with H1.2 and H1.4 being the predominant variants in most somatic cells [5]. The variants H1t, H1T2, H1oo and HILS1 are only found in germ cells [3,4]. Expression of H1x has only been investigated in a limited number of cell types [6]. H1 variants were shown to have a distinct nuclear localisation; for example, H1.2 seems to localize preferentially to euchromatic regions, whereas H1.4 is enriched in heterochromatic regions [7,8]. Whether somatic H1 variants have specific functions is subject to ongoing research [4]. Single knockout H1 variants in mice show upregulation of other H1 variants and relatively mild phenotypes [9]. However, knockout of three H1 variants leads to a 50% reduction of overall H1 amount and is embryonically lethal [10]. This highlights the potential importance of histone H1 in maintaining chromatin integrity, and suggests two possible functions of H1 variants: a general one redundant among H1 variants and related to the formation of higher order chromatin, and an individual, gene-specific one.

Covalent modifications of histones such as lysine methylation are involved in the regulation of all DNA-based processes. Methylation marks are catalysed by histone lysine methyl transferases (HKMTs) using S-adenosyl methionine (SAM) as the methyl group donor. The function of histone lysine methylation depends on the specific site and the methylation state (mono-, di- or trimethylated) [11]. However, not all HKMTs can catalyse the trimethylation state. G9a/KMT1C and the G9a-like protein 1 (Glp1/KMT1D) were initially described as enzymes that could mono- and dimethylate H3 on Lys9 (H3K9) in euchromatic regions, leading to repression of specific genes [12,13]. Knockout of either of these two enzymes is lethal at embryonic day (E9.5). Both knockouts result in drastic reduction of H3K9 mono- and dimethylation, leading to induction of specific genes. Furthermore, heterochromatin protein 1 (HP1), which binds to H3K9me2/me3, is relocalized in G9a/Glp1-deficient ES cells. Although G9a and Glp1 can independently methylate H3K9 *in vitro*, they form a heteromeric complex *in vivo*, explaining their similar phenotypes and the lack of redundancy [14]. Interestingly, some HKMTs have more than one target; for example, G9a was reported to methylate both H3 and H1.4 [15,16].

Most of the methylation marks characterized to date, are located in the N-terminal tails of the core histones H3 and H4. Posttranslational modifications of the linker histone H1 are less well studied. The best characterised H1 modification is phosphorylation [17,18]. This phosphorylation is cell cycle-dependent, reaching a maximum in M phase [17], and is mainly catalysed by cdk type kinases [17]. H1.4K26 methylation was the first methylation site discovered in H1. This methylation has been reported to be catalysed by Ezh2 as part of the PRC2 complex and G9a [16,19]. H1.4K26me2 is bound by HP1, leading to transcriptional repression, whereas phosphorylation of H1.4S27 impairs HP1 binding [20]. Technical improvements in mass spectrometry (MS) have lately led to the identification of new modification sites including methylation on various human H1 variants [18], but as yet their functions are unknown.

However, getting complete sequence coverage of H1 in MS analysis is very difficult, and several potential modified sites might be missed during the analysis. We sought to overcome this problem by using a candidate approach to identify HKMTs with activity on H1 and their target sites. We report for the first time that G9a and Glp1 are enzymes with activity towards the C-terminal tail of H1 both *in vitro *and *in vivo*, and we have mapped the methylation site on H1.2 to K187. We further show that methylation by G9a/Glp1 is H1 variant-specific, as G9a and Glp1 methylate preferentially H1.4K26 and H1.2K187 but not the corresponding lysines on H1.2 and H1.4, respectively. Additionally, these two methylation marks differ in their readout, as HP1 binds to methylated H1.4K26 but not to H1.2K187. Similarly, JMJD2D demethylates H1.4K26me but not H1.2K187me. Interestingly, the H1.2K187 methylation is constant over the cell cycle, in contrast to C-terminal H1 phosphorylation. Our data suggest that H1 variant-specific functions can be achieved through differential methylation of specific residues *in vivo*.

## Results

### K187H1.2 is a new methylation site in the C-terminus of H1.2

**Figure 1:**
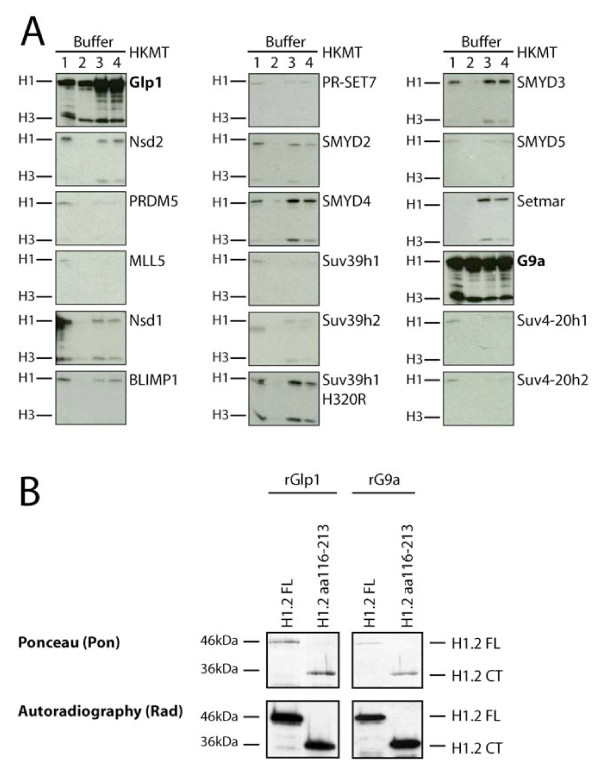
**G9a and its interaction partner Glp1 methylate H1. (a) **Candidate methylation assay on core nucleosomes and H1. Indicated HKMTs fused to GST (see also Methods) were used for methylation assays to identify enzymes methylating H1. Core nucleosomes (purified from HEK293) were mixed with H1 as substrates. All enzymes were tested in (lane 1) Tris, (lane 2) PBS, (lane 3) MAB and (lane 4) R methylation buffers. Incorporation of the methyl group from the donor adenosyl-L-methionine S-[methyl-3H] was detected by autoradiography (for a specificity control see Additional file 1). **(b) **Recombinantly expressed SET domains of Glp1 (610-917) and G9a (682-1000) and recombinant H1.2FL and H1.2CT were used for methylation assays. G9a and Glp1 methylate the H1.2 C-terminus (CT). Ponceau staining of the membrane (Pon, upper panels) and autoradiography (Rad, lower panel) are shown.

To find HKMTs specifically modifying histone H1 and to identify new methylation sites, we followed a candidate approach. A collection of described and potential HKMTs (all containing a SET domain) were recombinantly expressed and assayed for activity on HEK293 core nucleosomes and H1. Recombinant G9a and its interaction partner Glp1 had the strongest methylation activity towards histone H1 (Figure 1a), whereas the other enzymes did not modify H1 or were much less active on H1. Note that the enzymes used for these assays also methylate core histones, as reported previously [15,21], thus serving as a positive control (Figure 1a; see Additional file 1). We then focused our further analyses on the HKMTs most active on H1: G9a and Glp1.

Despite H1.2 being the most abundant H1 variant in many tissues and cell lines [5], the only characterized histone H1 methylation site is K26 in the N-terminus of H1.4 [19,20,22]. This prompted us to ask whether G9a and Glp1 can also methylate H1.2. Figure 1b shows that both enzymes can indeed methylate recombinant H1.2 *in vitro*. Interestingly, recent MS studies [18] suggested the existence of additional methylation sites, most likely located in the core region and the C-terminus of H1, which has been implicated in targeting and chromatin binding of H1. To investigate if G9a and/or Glp1 can methylate the C-terminus of H1.2, we assayed both enzymes on the H1.2 C-terminus (116-213). Both recombinant G9a and Glp1 (Figure 1b) and immunoprecipitated, eukaryotically expressed full-length G9a/Glp1 (data not shown) can methylate the C-terminus of H1.2, suggesting the presence of an additional G9a/Glp1-specific methylation site in the H1.2 C-terminus.

**Figure 2:**
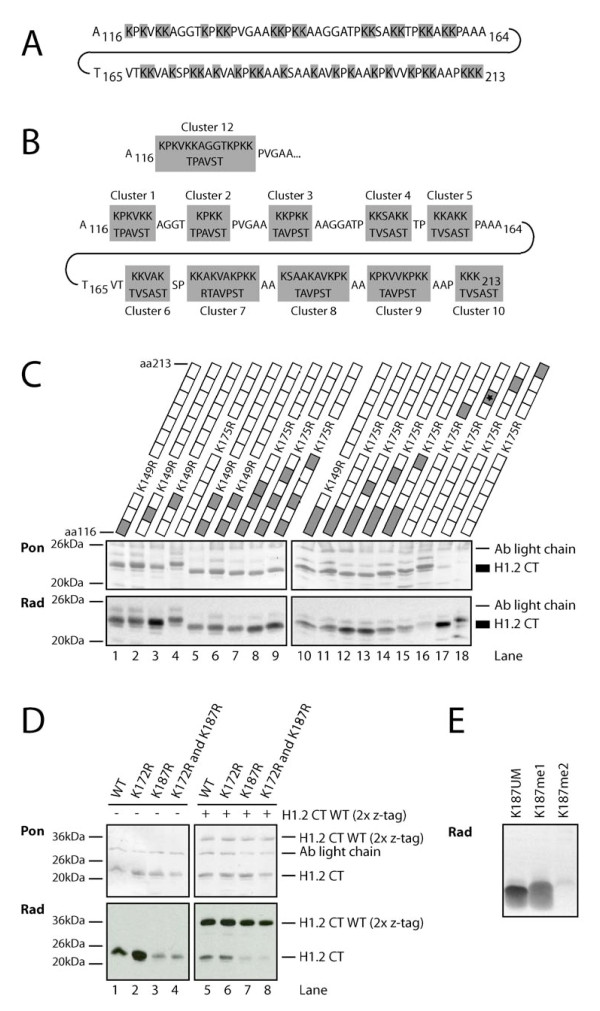
**G9a and Glp1 methylate H1.2K187**. **(a) **Amino acid sequence of the C-terminal tail of H1.2. Lysines as potential methylation sites are highlighted in grey. **(b) **Cluster mutation approach to map methylation sites in the C-terminal part with its 40 potential sites. Cluster definitions and their substituting amino acid sequence are depicted. All lysines in the C-terminal tail of H1.2 were classified into 10 clusters (grey rectangles). Cluster 12 is a fusion of clusters 1 and 2. (Upper) Wild-type H1.2 sequence; (lower) the substituting amino acids. **(c) **The methylated site is between amino acids 187 and 196. Methylation assay of the cluster mutation constructs with immunoprecipitated Glp1. Used constructs are schematically indicated on top. Grey rectangles, mutated clusters; white rectangles, wild-type clusters. In addition, all constructs carry a lysine to arginine mutation introducing an arginase C cutting site. (Upper panel) Ponceau (Pon) staining as a loading control. The different migration levels of the constructs are due to the substitutions and replacements of different numbers of uncharged and positively charged lysines by uncharged amino acids. Note a strong reduction in methylation in lane 16, corresponding to a cluster 8 mutation (mutated cluster containing K187 is indicated by asterix). **(d) **Methylation assay with wild-type H1.2CT, H1.2CT K172R, H1.2CT K187R, H1.2CT K172R and K187R and wild-type H1.2CT (2× z-tag) as substrates. Mutation of K187 results in a strong reduction of methylation by immunoprecipitated Glp1. (Upper panel) Pon; (lower panel) autoradiography (Rad). Wild-type H1.2CT (2× z-tag) was added to the methylation assays as an internal control of methyltransferase activity (lanes 5 to 8). **(e) **Methylation assays on peptides. Peptides containing unmodified K187, monomethylated K187 and dimethylated K187 and immunoprecipitated G9a were used for methylation assay. Rad is shown. Note that unmodified and monomethylated peptide were methylated to similar extents, whereas the dimethylated peptide was not methylated.

Next, we attempted to map this new methylation site in the C-terminus of H1.2. Because the C-terminus of H1 is extremely lysine-rich (a total of 40 lysine residues) (Figure 2a), a cluster mutation approach of the H1.2 C-terminus was first undertaken. All lysines were grouped into 10 clusters (Figure 2b) and replaced by non-lysine amino acids. These recombinantly expressed proteins were then used for *in vitro *methylation assays with immunoprecipitated, eukaryotically expressed Glp1 and G9a. As shown in Figure 2c, the H1 in which the cluster of amino acids 187 to 196 is mutated (lane 16) was markedly less methylated by Glp1 than the wild type C-terminus (lane 4) and all other constructs. This suggests that the main methylation site for Glp1 in the C-terminus resides in this amino acid stretch. Identical results were obtained for G9a (data not shown).

To identify the lysine residues methylated by G9a and Glp1, individual lysines in this cluster were mutated, starting with Lys187. Mutation of Lys187 to Arg resulted in almost complete loss of methylation activity of the C-terminus of H1.2 (Figure 2d, lanes 1 and 3), but not of other lysines in the cluster (data not shown). As a specificity control, Lys172, which is within a similar sequence motif (AKS) was also mutated; however, this mutation did not reduce methylation activity by Glp1 (Figure 2d, lane 2). To exclude the possibility that the lower methylation of the K187R mutant was due to inhibiting activities in the reaction, wild-type H1.2 CT (2× z-tag) was added as an internal control. Note that wild-type H1.2 CT (2× z-tag) becomes equally methylated in all the reactions (Figure 2d, lanes 5 to 8). Specificity was confirmed by tandem MS (MS/MS) analysis of the *in vitro *methylated H1.2 (data not shown). In parallel, peptides spanning amino acids 182 to 193 were used as substrates in methylation assays. Peptides unmethylated on K187 could be methylated by G9a (Figure 2e). For all these assays, identical results were obtained for Glp1 and G9a (data not shown). G9a and Glp1 had been previously reported to catalyse mono- and dimethylation [12,23]. Indeed, G9a and Glp1 can dimethylate a monomethylated K187 peptide but cannot trimethylate a dimethylated K187 peptide, again indicating that both enzymes can catalyse the mono- and dimethylation (Figure 2e, data not shown) as shown for H3K9 *in vitro *and *in vivo *[12,14,24]. Together, these data identify K187 as a new methylation site within the C-terminus of H1.2 and show that the HKMTs G9a and Glp1 can methylate this site *in vitro*.

### K187H1.2 is methylated by G9a and Glp1 *in vivo*

**Figure 3:**
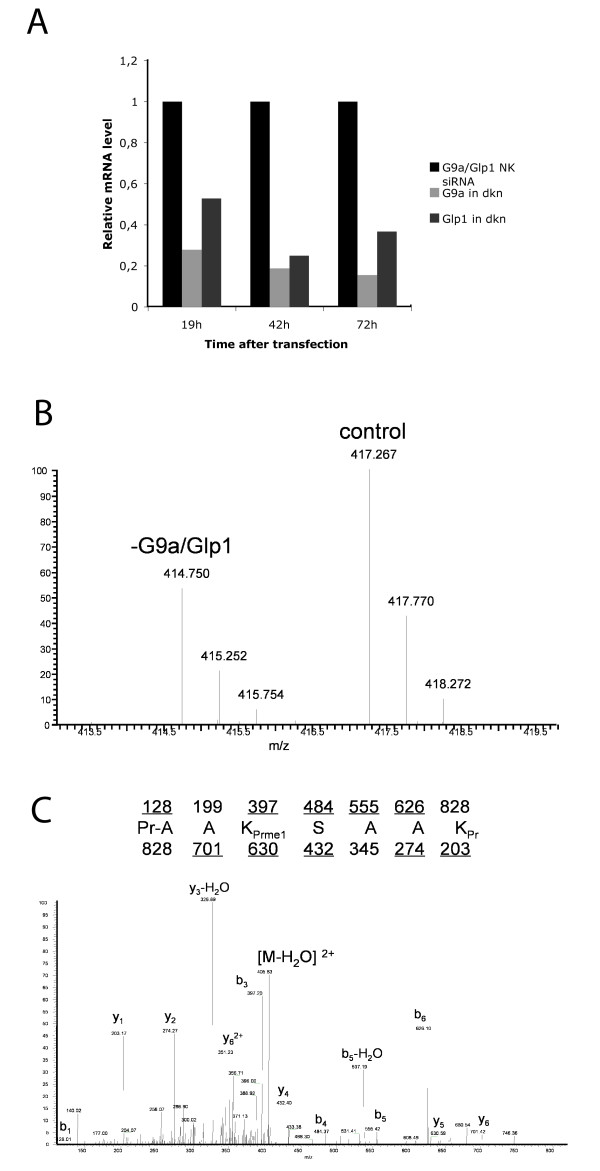
**G9a and Glp1 methylate H1.2K187 *in vivo***. **(a) **Reverse transcription PCR analysis of siRNA-transfected cells harvested at indicated time points. HEK293 cells were either transfected with a control siRNA (negative control; NK) or simultaneously with siRNAs against G9a and Glp1 (double knockdown; dkn). mRNA levels were normalised to β-actin expression. The mRNA levels of G9a and Glp1 in the control cells were set as 1 and levels in the double knockdown cells calculated as the ratio of this level (light grey, G9a; dark grey, Glp1). **(b) **MS analysis of H1.2 after knockdown. Full MS result showing the quantitative comparison of H1 peptides from control and G9a/Glp1 double knockdown samples. We observed an approximate twofold decrease in a peptide (414.750 m/z) from the G9a/Glp1 dkn sample compared with the control. **(c) **This peptide at 414.750 m/z was sequenced by MS/MS experiments and determined to be the peptide containing the K187 monomethylation mark. The dimethylation of H1.2K187 might have escaped our mass-spectrometric analysis by being below of our current detection threshold.

To confirm that G9a and Glp1 can also methylate K187 of H1.2 *in vivo*, RNA interference (RNAi) analysis was performed. HEK293 cells were used with either non-target negative control small interfering siRNA or simultaneously with two siRNAs against G9a and Glp1 to knock down both enzymes. At 42 hours and 72 hours after transfection of the siRNAs, a reduction in G9a RNA to approximately 20% and Glp1 to 30% was detected by RT-PCR (Figure 3a), and this knockdown was confirmed by western blotting (data not shown).

Histones were then acid-extracted from these cells and prepared by derivatization with D0- and isotopic D5-propionic anhydride for quantitative proteomic analysis. After derivatization, control and knockdown samples were mixed equally; they could be distinguished because of a mass difference corresponding to a 5 Da shift. Two sets of peaks corresponding to a 2.5 Da shift were seen, which is consistent with D0- and D5-propionyl labelling for a 2+ charged peptide. The D0 labelled peptide from the G9a/Glp1 knockdown sample was decreased by approximately two fold compared with the control (Figure 3b), and MS/MS analysis of this D0 peak (Figure 3c) confirmed that this peptide contained the sequence pr-AAK_prme_SAAK_pr _(pr = propionyl amide, +56 Da). To control for enzyme specificity, the effect of the knockdown on other histone modifications was analysed, and as previously described [16], a reduction in H1.4K26me and H3K9me was seen, whereas H3K27me and H4K20me did not change (Additional file 2). In summary, this demonstrates that K187 of H1.2 is methylated *in vivo *and that G9a and Glp1 can mediate this methylation.

### K187 methylation by G9a/Glp1 is H1 variant-specific

**Figure 4:**
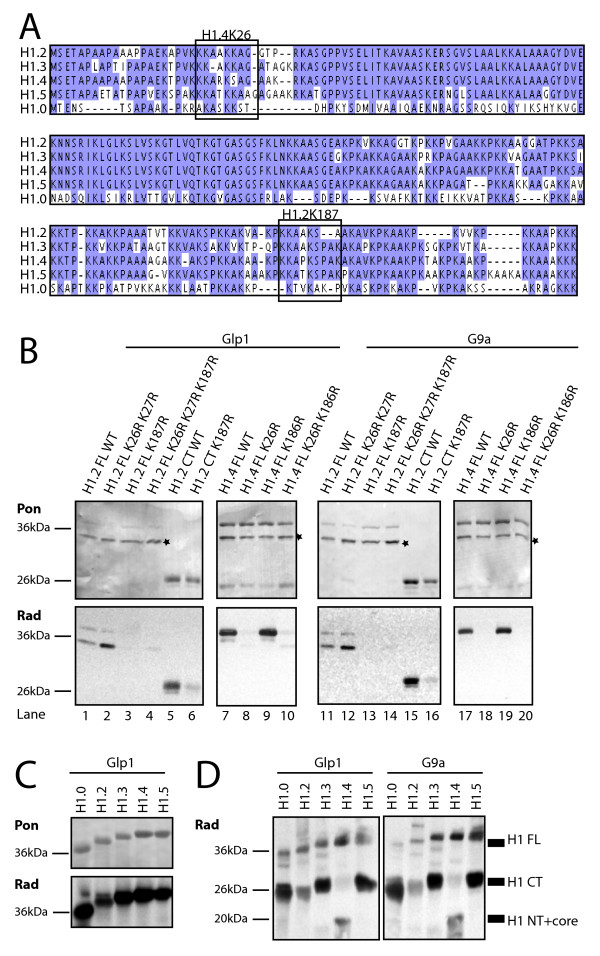
**Variant-specific methylation of H1 by G9a/Glp1**. **(a) **Alignment of H1 variants H1.2, H1.3, H1.4, H1.5 and H1.0. Amino acids identical in at least three variants are highlighted in blue. The regions around H1.4K26 and H1.2K187 are boxed. **(b) **H1.4K26 and H1.2K187 are G9a and Glp1 targets. Methylation assay on indicated H1.2 and H1.4 mutants using (left) immunoprecipitated Glp1 and (right) G9a. Note that the high expression of H1 led to partial N-terminal degradation of some constructs in bacteria. (Upper panels) Ponceau (Pon) staining: *degradation fragments. (Lower panels) Autoradiography (Rad). **(c) **H1.2, H1.3, H1.4, H1.5 and H1.0 were methylated by G9a and Glp1. Methylation assays on recombinant human H1 variants using immunoprecipitated G9a and Glp1. (Upper panels) Ponceau (Pon) staining; (lower panels) autoradiography (Rad). **(d) **Methylation assays on human recombinant H1 followed by chymotrypsin digestion, which separates the N-terminus and core from the C-terminus. Only the N-terminus and core of H1.4 is a major G9a/Glp1 target whereas H1.2, H1.3, H1.5 and H1.0 are methylated by G9a/Glp1 preferentially in the C-terminal part. Autoradiography (Rad) is shown (loading control in Additional file 3).

Up to this point, our studies had been focused on methylation of the H1.2 C-terminus. Because it had recently been described that G9a can methylate K26 on H1.4 [16], we then investigated whether H1.2 can also be methylated by G9a or Glp1 at K26 or at the neighbouring K27 (Figure 4a). Both lysines in full-length H1.2 were mutated, and methylation assays performed *in vitro*. Whereas mutation of K187 resulted in a strong decrease in methylation (Figure 4b; lanes 1 and 11 versus lanes 3 and 13), mutation of K26 and K27 did not impair H1.2 methylation by Glp1 or G9a (Figure 4b, lanes 2 and 12). By contrast, mutation of H1.4K26 resulted in an almost complete loss of methylation (Figure 4b, lanes 8 and 18) whereas mutation of K186 (corresponding to K187 in H1.2) did not affect the methylation of H1.4 by Glp1 and G9a (Figure 4b, lanes 9 and 19). Thus, G9a/Glp1 methylated H1 in a variant-specific manner, preferentially targeting the N-terminus of H1.4, but the C-terminus of H1.2.

These results prompted us to investigate whether the other major H1 variants are methylated by G9a/Glp1 on the N- or C-terminus. First, *in vitro *methylation assays were performed, using recombinant H1.3, H1.5, and the more divergent variant H1.0; the latter is mainly expressed in terminally differentiated cells. All of the H1 variants analysed could be methylated by G9a/Glp1 (Figure 4c). To distinguish between N- and C-terminal methylation, H1 was digested after the methylation reaction with chymotrypsin, which cleaves the H1 C-terminus at position F105 [17]. G9a/Glp1 methylated preferentially the N-terminus of H1.4, whereas they methylated preferentially the C-termini of other variants (Figure 4d).

Together, these data demonstrate that G9a and Glp1 methylate residues within H1 in a variant-specific fashion. H1.4 is specifically methylated at the N-terminus at K26 and H1.2 at the C-terminus at K187, but not the corresponding lysines on H1.2 and H1.4, respectively. Furthermore, the C-terminus of H1 is the main target for G9a/Glp1-mediated methylation in most somatic H1 variants.

### H1.2K187 methylation is cell cycle-independent

**Figure 5:**
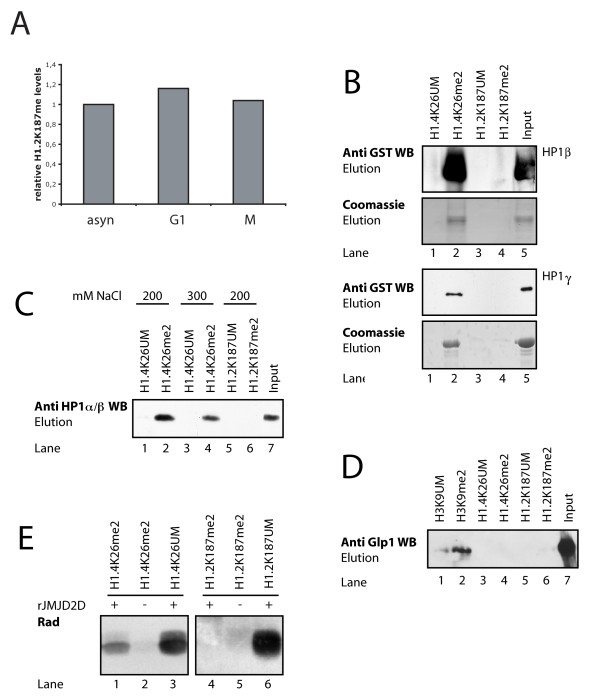
**H1.2K187 methylation is cell cycle-independent and HP1 binds only to methylated H1.4K26**. **(a) **H1.2K187me is cell cycle-independent. Quantitative MS analysis of H1 extracted from unsynchronized HEK293 cells and synchronized cells in G1 and M phase. A selected ion monitoring experiment was used on the ion 414.745 m/z (corresponding to H1K187 methylation) to monitor the levels of methylation. Normalisation was done relative to unsynchronized cells. **(b) **Recombinant HP1 binds to H1.4K26me2 but not to H1.2K187me2. Peptide affinity purification of recombinant HP1β and γ (GST fusion protein). The upper panels show the anti GST WB of the elution and lower panels the Coomassie staining of eluted fraction. **(c) **Endogenous HP1 binds to H1.4K26me2 but not H1.2K187me2. Western Blot analysis of peptide affinity purification of nuclear extracts (HeLa extracts) with HP1α/β specific antibody. **(d) **Glp1 does not bind to H1.4K26me2 or H1.2K187me2. Western Blot analysis of peptide affinity purification of nuclear extracts (HeLa extracts) with Glp1 specific antibody. **(e) **JMJD2D demethylates H1.4K26me2 only. Methylation assay with G9a and Glp1 after demethylation of peptides by JMJD2D (Rad).

Histone H1 phosphorylation is also mainly localised to the C-terminus. This C-terminal H1 phosphorylation is highly cell cycle-regulated [17], reaching a maximum in G2/M. Based on this, we investigated if H1.2K187 is also cell cycle-regulated. Therefore, HEK293 cells were synchronised and H1 isolated at different time points for quantitative MS analysis. H1.2K187 methylation levels stayed relatively constant during the cell cycle (Figure 5a), indicating that H1.2 C-terminal phosphorylation and H1.2K187me may act independently of each other.

### H1.2K187 and H1.4K26 have different downstream readers

To identify novel, methylation-dependent H1 binding proteins, we followed an unbiased approach initially, using peptide affinity purifications. We did not detect any novel H1 methylation-specific binding proteins (data not shown), thus we switched to a candidate approach. We showed previously [20] that HP1 binds specifically to methylated K26 of H1.4. Because H1.2K187 and H1.4K26 are methylated by the same enzymes, we investigated if HP1 could also bind to methylated H1.2K187. Recombinant HP1β and γ were expressed and assayed for binding to K187me2 in peptide affinity purifications. The recombinant HP1β and γ bound to methylated H1.4K26me2 (Figure 5b, lane2) but not to H1.2K187me2 (lane 4). To confirm this with endogenous proteins, the peptide affinity purifications were repeated using HeLa nuclear extract as HP1 source. There was specific binding of endogenous HP1 to H1.4K26me2 (Figure 5c, lane 2), but not to H1.2K187me2 (Figure 5c, lane 6). These data indicate selective recognition by HP1: H1.4K26me2 is specifically bound by HP1 whereas H1.2K187me2 is not.

The ankyrin repeats of G9a and Glp1 have been shown to bind specifically to H3K9me1/2 and it has been suggested that this binding could be involved in the recruitment of the enzymes. Therefore, we investigated if they could also bind to methylated H1. We did not observe any specific binding of Glp1 (Figure 5d) (or G9a, data not shown) to either H1.4K26me2 or H1.2K187me2, arguing against a binding of G9a/Glp1 to its H1 substrate sites.

To gain further insight into regulation of K187 methylation we investigated whether the jumonji-C type histone demethylase JMJD2D/KDM4 (which can reverse dimethylation to monomethylation) could demethylate H1.2K187me2, as shown for H1.4K26me2 [16]. H1.2K187me2 and H1.4K26me2 peptides were first demethylated and then assayed for remethylation with G9a and Glp1. This assay is indirect but very sensitive, as low levels of demethylation are sufficient to allow remethylation. Incubation of the dimethylated H1 peptides with recombinant JMJD2D allowed remethylation by G9a/Glp1 of the H1.4K26 peptide (Figure 5e, lane 1) but not of the H1.2K187 peptide (lane 4). Thus H1.4K26me2 but not H1.2K187me2 can be demethylated by JMJD2D.

Together, these results show that despite being methylated by the same HKMTs, H1.4K26 and H1.2K187 methylation are read and removed by different proteins, suggesting novel variant-specific functions.

## Discussion

Our data show that K187 in histone H1.2 is a new target of HKMTs G9a and its interaction partner Glp1 *in vitro *and *in vivo*, as confirmed by mutation analysis, knockdown experiments and MS analysis. To our knowledge, this is the first characterisation of a methylation site and its corresponding methylating enzymes in the C-terminus of histone H1. G9a and Glp1 were found to be in heteromeric complexes and to share high sequence homology, and in the case of histone H3, both contribute to global H3K9 mono- and dimethylation [14]. We show here that a similar situation occurs for H1, where both enzymes target identical lysines.

Interestingly, both G9a and Glp1 were able to methylate H1 in a variant-specific way. In H1.4, the main methylation site is K26 in the N-terminus, whereas in H1.2 (and the other main somatic H1 variants H1.0, H1.3 and H1.5) it is the C-terminus that is preferentially methylated by G9a and Glp1. Methylation of H1.2 at K26 had previously been identified only at low levels by MS in certain mouse tissues, but according to our data this seems not to be a G9a target. Indeed, H1.2K26 can be methylated by other enzymes such as Ezh2 ([18] and our unpublished data). Furthermore, H1.4K26me2 is specifically bound by all HP1 isoforms, but K187me2 is not, and only H1.4K26me2 is demethylated by JMJD2D. Both of these features thus distinguish these two methylation marks.

In our MS analysis we detected monomethylation of H1.2K187 *in vivo*, but dimethylation of H1.2K187 might have escaped our MS analysis if it was below our current detection threshold. Both enzymes have been shown to dimethylate H3K9 and H1.4K26 both *in vitro *and *in vivo *[16,24]. In line with this, our assays showed that G9a and Glp1 methylate unmodified and monomethylated K187 peptides, demonstrating dimethylation activity.

To date, the only characterised modification of the H1 C-terminus is phosphorylation. The C-terminal tail of H1 can be highly phosphorylated [25] on several serine and threonine residues. This phosphorylation is mainly catalysed by cdk type kinases [26,27], and is highly cell cycle-dependent, reaching maximum levels in G2/M phase. Interestingly, in contrast to phosphorylation, H1.2K187 methylation levels remain steady throughout the cell cycle, suggesting that only H1 C-terminal phosphorylation, but not H1.2K187 methylation, has a global role in mitotic chromosome condensation [28]. In a recent MS analysis [3,4,18,29] a phosphorylation site, S188, was mapped next to K187 in H1.4 but not H1.2. Although it is tempting to speculate that S188 phosphorylation might block K187 methylation in H1.4, there are strong arguments against this. First, the C-terminus of recombinant, unphosphorylated H1.4 is not (or only very weakly) methylated by G9a/Glp1 and second, only the neighbouring phosphorylation is cell cycle-dependent.

Whether H1 variants have specific functions is a matter of an ongoing debate [3,4,29]; however, there are several lines of evidence pointing towards specific functions. Fluorescence recovery after photobleaching experiments showed that different H1 variants span a broad range of recovery times, owing to significant differences in their binding affinity to chromatin [3,4,29]. The recovery time depends on the length of the C-terminal tail, on the density of positively charged amino acids, and on the number and distribution of phosphorylation sites within it. H1.2 has one of the shortest tails and the fastest recovery time, whereas H1.4 shows higher affinity for chromatin with longer residence times [8]. Recently, differences in the binding affinities of mammalian H1 variants have also been shown both *in vitro *[30] and *in vivo *[31]. Additionally, a preferential location of H1 variants to different genomic regions was described [8]. Both the differences in the dynamics and the reported variations in global nuclear distribution patterns suggest that H1 variants such as H1.2 and H1.4 (the major H1 variants in many somatic cells) could have specific functions. This is supported by our findings that different methylation sites targeted by the same enzymes on distinct H1 variants have specific readouts.

In addition to G9a/Glp1, H1.4K26 can be methylated by Ezh2 as part of the polycomb repressive complex 2 (PRC2) [22]; however, the relative *in vivo *contribution of these enzymes is currently unclear. Inhibition of G9a and Glp1 results in a strong decrease in H1.4K26 methylation [16], suggesting that G9a/Glp1 might be the main enzymes catalysing this modification. This is in line with our knockdown experiments showing that reduced levels of G9a and Glp1 drastically affect H1.4K26me (see Additional file 2).

Pinpointing a cellular phenotype to histone modifications in mammals is not straightforward, because amino acid substitutions cannot be applied by genetic approaches and also because the enzymes catalyzing modifications often modify multiple proteins. The phenotype of the G9a/Glp1 knockout is embryonically lethal [24]; however, it is unclear whether this is due to changes in the histone modifications or in other G9a/Glp1 targets [32]. In an attempt to investigate the cellular function of H1.2K187, we made stable cell lines expressing tagged wild-type H1.2K187 and a H1.2K187R mutant that cannot be methylated. Because depletion of H1.2 has been shown to result in cell-cycle arrest in G1 phase [31], we checked particularly for possible effects on the cell cycle; however, we did not detect any major effect on the cells or on cell-cycle progression, which is in line with H1.2K187 methylation levels remaining rather steady throughout the cell cycle.

Interestingly, H1.4K26 methylation by G9a has been shown to increase the residence time of H1.4 on chromatin [16], whereas the more 'dynamic' H1.2 is not methylated by G9a at this site. Because both G9a and Glp1 are important for H3K9 methylation and silencing of euchromatic genes [14,24], and because H1.2 has been found to be enriched in euchromatic regions [7,8], we speculate that H1.2K187 methylation might have a role in gene silencing in these regions. By contrast, H1.4 has been found to be enriched in heterochromatic regions [7], and methylated H1.4K26 but not H1.2K187 can be specifically bound by HP1 [20], a main component of heterochromatin. This might argue for a role of H1.4K26 methylation in transcriptional repression in heterochromatin. It will be interesting in the future to determine the biological functions and the localisation patterns of the H1 variants and their corresponding modifications at a genome-wide level. To date, in spite of many attempts and different approaches, we have been unable to raise a satisfactory antibody with unique specificity for methylated H1.2K187 that could be used in *in vivo *experiments.

It has been reported that the ankyrin repeats of G9a and Glp1 recognise H3K9me1/2 [33], and suggested that they also bind to H1.4K26me1/2 [16], contributing to their recruitment to chromatin. We do not observe binding of G9a or Glp1 to methylated H1.4K26 or H1.2K187 peptides, suggesting a different recruiting mechanism from that for H3K9me1/2. Moreover, recruitment of G9a/Glp1 to H3K9me1/2 could result in the spreading of H3K9 methylation mark, whereas our data suggest that such a mechanism is less likely for H1. This is in line with the globally low levels of H1 methylation, compared with H3K9me1/2; however, binding of the ankyrin repeats to H3K9me1/2 could tether G9a/Glp1 and then direct H1 methylation.

## Conclusions

In conclusion, we have identified and characterised a novel methylation site in the C-terminus of histone H1 (H1.2K187). We have shown that the HKMTs G9a and its interacting partner Glp1 are the enzymes performing this methylation *in vitro *and *in vivo*, resulting in the identification of the first enzymes that methylate the H1 C-terminus and of a new G9a/Glp1 target. Furthermore, we have demonstrated that the HKMTs G9a and Glp1 can methylate histone H1 in a variant-specific manner in the N- or C-terminus. To our knowledge, this is the first example of variant-specific histone modifications by the same enzymes. Additionally H1.4K26me and H1.2K187me also differ in their readout, clearly distinguishing them functionally.

## Methods

### Cluster mutation PCR

For the exchange of lysine clusters, two-step mutagenesis PCR was used. Mutation primers consisted of the 18 replacing bases and approximately 20 bases complementary to the template. In the first PCR, DNA fragments covering the 5' end to the mutation site and the mutation site to the 3' end were amplified separately in two reactions. In the second PCR, the two products were mixed and primers flanking the 5' and 3' ends were used to amplify the complete mutated DNA fragment.

### Protein expression and purification

Recombinant human HKMTs ((numbers are amino acid residues) Glp1 610-917; Nsd2 926-1210; PRDM5 3-429; MLL5 294-481; Nsd1 1700-1987; BLIMP1 105-323; PR-SET7 FL; SMYD2 FL; SMYD4 FL; Suv39h1 82-412; Suv39h2 157-477; Suv39h1 82-412 H320R; SMYD3 FL; SMYD5 FL; G9a 682-1000; Suv4-20h1 1-384; Suv4-20h2 1-280; plasmids obtained from T. Jenuwein) were expressed as glutathione S-transferase (GST) fusion proteins in bacteria using standard techniques and purified (glutathione Sepharose^® ^4B; GE Healthcare, Piscataway, NJ, USA).

Affinity purification of bacterially expressed 2× z-tagged and histidine (His)-tagged H1 was performed (HIS-Select Nickel Affinity Gel; Sigma, St Louis, MO, USA). C-terminally His-tagged recombinant histone H1 was extracted from bacteria with 0.83 M perchloric acid (PCA) and then neutralized by addition of Tris/HCl pH 9.5. Further purification was achieved using nickel affinity gel (Sigma).

For eukaryotic expression of human G9a and Glp1, HEK293 cells were transiently transfected with plasmids encoding full-length FLAG-tagged G9a or Glp1 using the calcium-phosphate method and harvested 72 hours after transfection. Purification was performed using Anti-FLAG M2 agarose (Sigma).

His-tagged recombinant human JMJD2D (1-350; plasmid obtained from Y. Shi) was expressed in bacteria using standard techniques and purified with nickel affinity gel. Human FL HP1β and human FL HP1γ (plasmids obtained from T. Kouzarides) were expressed as GST fusion proteins in bacteria using standard techniques and affinity purified (glutathione Sepharose^® ^4B; GE Healthcare). Full-length recombinant H1 variants were obtained from Calbiochem -Novabiochem Corp. (San Diego, CA, USA).

### Purification of endogenous nucleosomes and H1

Approximately 3 × 10^7 ^HEK293 cells were swollen in hypotonic buffer (20 mM HEPES pH 7.0, 20 mM NaCl, 5 mM MgCl_2_), and plasma membranes were destroyed by homogenizing in a dounce homogenizer. After centrifugation, the pellet was resuspended in hypotonic buffer with a final concentration of 0.5% NP-40 (Nonidet P-40 (Fluka AG, Buchs, Switzerland); to lyse nuclei. Chromatin was pelleted by centrifugation, resuspended in isolation buffer (10 mM Tris/HCl pH 7.4, 1.5 mM MgCl_2_, 1 mM CaCl_2_, 0.25 M sucrose) and optical density at 260 nm (OD_260_) was measured. Micrococcal nuclease (10 U per 50 μg DNA) was added, and the samples were incubated at 37°C for 1 hour. Mono- and dinucleosomes were extracted twice with lysis buffer (10 mM Tris/HCl pH 6.85, 5 mM EDTA) and separated by centrifugation for 14 hours at 280 000 *g *on a 5% to 40% sucrose gradient (20 mM HEPES pH 7.6, 0.5 mM MgCl_2_, 5% to 40% sucrose).

For endogenous H1 extraction, HEK293 cells were lysed in Triton extraction buffer (1 × phosphate-buffered saline (PBS) with 0.5% triton ×100) to remove the cytosol. After pelleting of nuclei, H1 was extracted with 0.83 M PCA.

### *In vitro *methylation assay

HKMT assays were carried out as previously described [34]. Briefly, in a total volume of 25 μl, 2-10 μl of purified full-length G9a or Glp1 or recombinant GST fusion enzymes were mixed with 2 μg of recombinant histone H1 or 10 μg of peptides as substrate (H1.2K187 peptides PKKAAK_187_SAAKAVGC with K187 either unmodified (UM), monomethylated (me1) or dimethylated (me2), H1.4K26 peptides VKKKARK_26_SAGAAKGC with K26 either unmodified or dimethylated) (peptides obtained from CloneStar Peptide Services (Brno, Czech Republic) and GeneCust Europe (Dudelang, Luxembourg)). Reactions were carried out in Tris (20 mM Tris/HCl pH 9.0), phosphate (10 mM Na_2_HPO_4_, 2 mM KH_2_PO_4_, 2.7 mM KCl, 137 mM NaCl, pH 7.2), R (50 mM Tris/HCl pH 8.5, 5 mM MgCl_2_) or Methylation Assay Buffer (MAB) (50 mM Tris/HCl pH 8.5, 20 mM KCl, 10 mM MgCl_2_, 0.25 mM sucrose) buffers at 30°C for 1 hour. H1 was then separated using SDS-PAGE with 15% to 18.7% gels, and peptides were resolved in 16% Schägger Jagow gels. Proteins and peptides were transferred to nitrocellulose membranes and exposed to film (BioMax MS; Kodak).

### *In vitro *demethylation assay and subsequent remethylation assay

For the demethylation assay, 20 μl of purified recombinantly expressed JMJD2D and 25 μg of peptide were mixed in reaction buffer (50 mM Tris/HCl pH 7.4, 1 mM α-ketoglutarate, 2 mM ascorbate and 70 μM Fe2+(NH4)2(SO4)2), and demethylation was performed at 37°C for 6 hours. For subsequent remethylation, 15 μl of the demethylation assay mixture were added to 1 μl of 1 M Tris/HCl pH 9.5, 9.4 μl of mixed purified G9a and Glp1, 0.6 μl 0.5 M EDTA (final concentration 10 mM) and 4 μl 5× R methylation buffer, and then incubated at 32°C for 1 hour. Peptides were separated in 16% Schägger Jagow gels, transferred to nitrocellulose membrane and exposed to film (BioMax MS).

### Knockdown of G9a and Glp1 and reverse transcriptase PCR

Briefly, 4 × 10^6 ^HEK293 cells were plated in a 145 mm dish. The following day, the cells were simultaneously transfected according to the manufacturer's instructions, using a mix containing 100 μl of 10 mM anti-Glp1 small interfering (si)RNA (AACGAAGAATGGGAACCTATA) (Qiagen, Valencia, CA, USA), 100 μl anti-G9a siRNA (CACCATGAACATCGATCGCAA) (Qiagen) and 100 μl of lipofectamine for double knockdown. Control cells were transfected with 200 μl 10 mM negative siRNA (AllStars; Qiagen). The mRNA levels of cells harvested after 19, 42 and 72 hours were analysed by reverse transcriptase PCR using a commercial kit (RevertAid Minus First Strand cDNA Synthesis Kit; Fermentas Inc., Glen Burnie, MD, USA) and the following primers: CTGACACAGAGGACAGGAAGC and TCTCGAACTTCTCTGGGATCTT for Glp1; TCCGACAGCAAGTCTGAAGTT and TGACTGATTCCCTGACTCCTC for G9a; CGGTTGGCCTTGGGGTTCAGGGGG and ATCGTGGGGGCGCCCCAGGCACCA for β-actin (used as control). For MS analysis, cells were harvested after 72 hours.

### Peptide affinity purification

Peptides were coupled to a gel (SulfoLink Coupling Gel; Pierce, Rockford, IL, USA) via their C-terminal cysteine according to the manufacturer's instructions. Recombinant HP1β and γ or HeLa nuclear extracts were added to 20 μl of coupled beads and incubated for 90 minutes at 4°C. Beads were washed three times with IPH-X buffer (20 mM Tris/HCl pH 8.0, 0.5% NP-40, NaCl 250 mM or as indicated) before elution and separation on 15% SDS-PAGE gels. As indicated, binding was detected by either Coomassie staining or immunostaining using HP1 -specific antibody (10478; Abcam, Cambridge, MA, USA) or Glp1-specific antibody (D220-3; MBL International, Woburn, MA, USA).

### Mass spectrometry

Acid-extracted histones were diluted in 100 mM ammonium bicarbonate buffer (pH 8.0) and digested with trypsin at a protein:substrate ratio of 15:1 for 8 hours at 37°C. The reaction was quenched by acidification to pH 3.0 with glacial acetic acid and freezing. The digestion was then reacted with either D0- or D5-propionic anhydride [35] for chemical derivatization labelling and incorporation of a stable isotope label for quantitative comparison as previously described (Plazas-Mayorca *et a*l., submitted for publication). Samples were separated by online reverse-phase liquid chromatography followed by analysis in an orbitrap mass spectrometer operated in the data-dependent mode as previously described [36]. All spectra were manually verified.

### Cell-cycle synchronization

Exponentially growing HEK293 cells were synchronised in M phase by treatment with 300 ng/ml nocodazole for 18 h. Cells were enriched in G1/G0 phase by serum starvation in DMEM containing 0,5% FCS for 48 h. Cell-cycle distribution was checked by propidium iodide staining and fluorescence activated cell sorting.

### Chymotrypsin digestion

For chymotrypsin digestion, 4 μl of 3 M Na acetate pH 4.8 and 60 ng chymotrypsin (A-Chymotrypsin Type I-S; Sigma) were added to 25 μl of methylation assay, and digestion was performed at 25°C for 20 minutes. Fragments were separated in 18.7% SDS-PAGE gels, transferred to nitrocellulose membrane and exposed to film (BioMax MS).

## Competing interests

The authors declare that they have no competing interests.

## Authors' contributions

TW carried out most of the experimental work with the help of SH, UZ, AI, MD and PT. BMZ and BAG performed the mass-spectrometrical analysis. TW, SD and RS participated in the design of the study and prepared the manuscript. All authors read and approved the final manuscript.

## Supplementary Material

Additional file 1 **Figure S1 - Recombinant PRSET7 and Suv4-20h1 methylate H4**. (a) Coomassie staining of membrane with histones used for Figure 1a. (b) Autoradiography of HKMT assay with PRSET7 and Suv4-20h1 is shown as specificity control of our assays. Click here for file

Additional file 2 **Figure S2 - Quantitative mass spectrometry analysis of G9a/Glp1 knockdown versus control samples**. Full mass spectra showing peptides from (a) H4K20me2, (b) H3K27me2, (c) H3K9me2 and (d) H1.4K26me1. No changes in H4K20me2 or H3K27me2 were detected, but decreases in H3K9me2 and H1.4K26me1 were seen in the G9a/Glp1 knockdown samples. Click here for file

Additional file 3 **Figure S3 - Loading control of digested H1 variants**. Coomassie loading control for Fig 4D. H1 variants were methylated by G9a/Glp1 and afterwards digested by Chymotrypsin. One part of the samples was loaded on an SDS gel and afterwards transferred to a nitrocellulose membrane for autoradiography (Fig 4d), the other part of the samples was loaded on an SDS gel and stained with Coomassie to serve as a loading control. Click here for file
