# Supplementary figures and images for: Histone H1 variant-specific lysine methylation by G9a/KMT1C and Glp1/KMT1D
Source: Epigenetics Chromatin. 2010 Mar 24;3:7. doi: 10.1186/1756-8935-3-7 (PMC2860349; doi:10.1186/1756-8935-3-7)

**A**

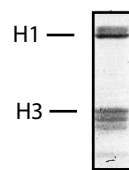

**B**

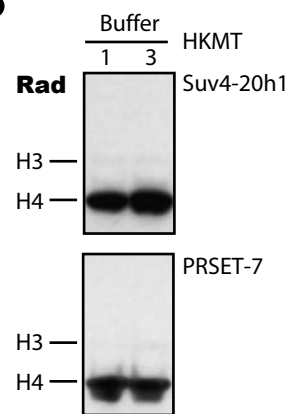

Supplementary Figure 1

Supplement: Additional file 1 — Figure S1 - Recombinant PRSET7 and Suv4-20h1 methylate H4. (a) Coomassie staining of membrane with histones used for Figure 1a. (b) Autoradiography of HKMT assay with PRSET7 and Suv4-20h1 is shown as specificity control of our assays. [file 1756-8935-3-7-S1.PDF]

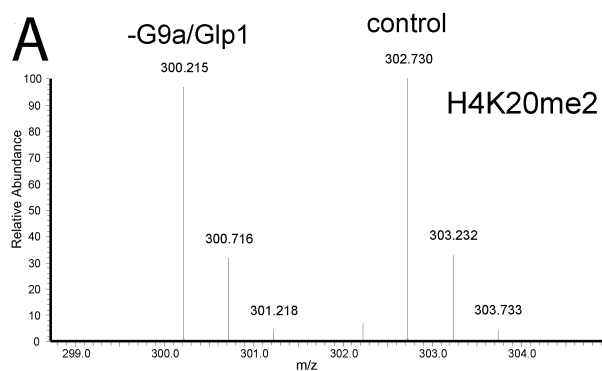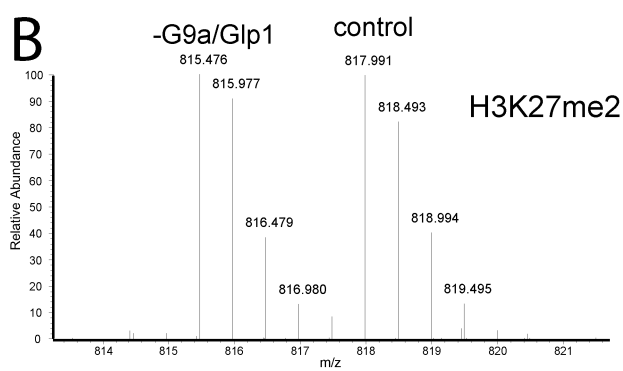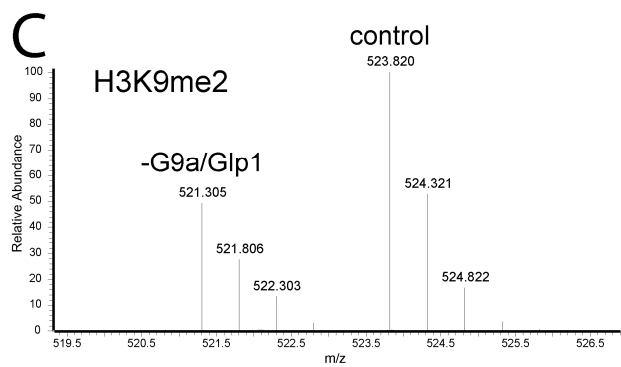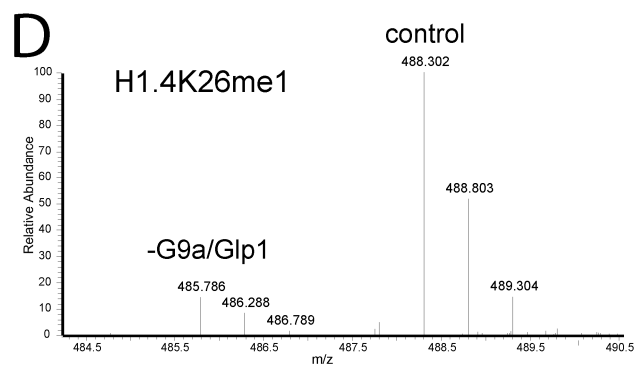

Supplementary Figure 2

Supplement: Additional file 2 — Figure S2 - Quantitative mass spectrometry analysis of G9a/Glp1 knockdown versus control samples. Full mass spectra showing peptides from (a) H4K20me2, (b) H3K27me2, (c) H3K9me2 and (d) H1.4K26me1. No changes in H4K20me2 or H3K27me2 were detected, but decreases in H3K9me2 and H1.4K26me1 were seen in the G9a/Glp1 knockdown samples. [file 1756-8935-3-7-S2.PDF]

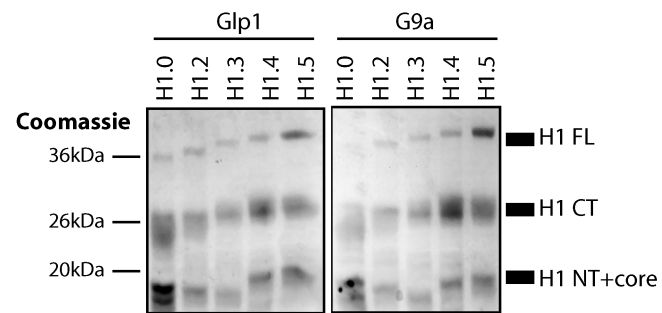

Supplementary Figure 3

Supplement: Additional file 3 — Figure S3 - Loading control of digested H1 variants. Coomassie loading control for Fig 4D. H1 variants were methylated by G9a/Glp1 and afterwards digested by Chymotrypsin. One part of the samples was loaded on an SDS gel and afterwards transferred to a nitrocellulose membrane for autoradiography (Fig 4d), the other part of the samples was loaded on an SDS gel and stained with Coomassie to serve as a loading control. [file 1756-8935-3-7-S3.PDF]
